# Exposure to Aldehyde Cherry e-Liquid Flavoring and Its Vaping Byproduct Disrupt Pulmonary Surfactant Biophysical Function

**DOI:** 10.1021/acs.est.3c07874

**Published:** 2024-01-08

**Authors:** Alexia Martin, Carmelo Tempra, Yuefan Yu, Juho Liekkinen, Roma Thakker, Hayoung Lee, Berta de Santos Moreno, Ilpo Vattulainen, Christos Rossios, Matti Javanainen, Jorge Bernardino de la Serna

**Affiliations:** † National Heart and Lung Institute, Imperial College London, Sir Alexander Fleming Building, London SW7 2AZ, U.K.; ‡ Institute of Organic Chemistry and Biochemistry, Czech Academy of Sciences, Prague 6 160 00, Czech Republic; § Department of Physics, University of Helsinki, Helsinki 00560, Finland; ∥ Institute of Biotechnology, University of Helsinki, Helsinki 00790, Finland

**Keywords:** e-cigarettes, vaping, lung surfactant, inhalation toxicology, mechanistic toxicology, exposure and human health

## Abstract

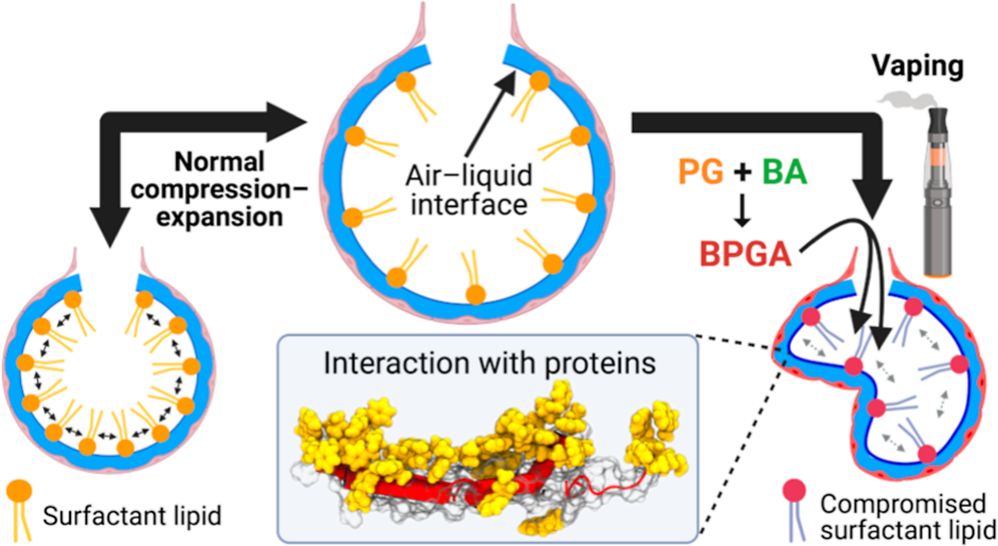

Over the past decade, there has been a significant rise in the use of vaping devices, particularly among adolescents, raising concerns for effects on respiratory health. Pressingly, many recent vaping-related lung injuries are unexplained by current knowledge, and the overall implications of vaping for respiratory health are poorly understood. This study investigates the effect of hydrophobic vaping liquid chemicals on the pulmonary surfactant biophysical function. We focus on the commonly used flavoring benzaldehyde and its vaping byproduct, benzaldehyde propylene glycol acetal. The study involves rigorous testing of the surfactant biophysical function in Langmuir trough and constrained sessile drop surfactometer experiments with both protein-free synthetic surfactant and hydrophobic protein-containing clinical surfactant models. The study reveals that exposure to these vaping chemicals significantly interferes with the synthetic and clinical surfactant biophysical function. Further atomistic simulations reveal preferential interactions with SP-B and SP-C surfactant proteins. Additionally, data show surfactant lipid–vaping chemical interactions and suggest significant transfer of vaping chemicals to the experimental subphase, indicating a toxicological mechanism for the alveolar epithelium. Our study, therefore, reveals novel mechanisms for the inhalational toxicity of vaping. This highlights the need to reassess the safety of vaping liquids for respiratory health, particularly the use of aldehyde chemicals as vaping flavorings.

## Abstract 2

Minimal research dwells on inhaled vaping liquid mechanistic toxicity. This study reports how a common vaping ingredient and its byproduct compromise lung surfactant function at the molecular level with implications for human health.

## Introduction

Ever since their introduction to the market in 2006,^1^ vaping devices have rapidly grown in popularity. This is largely due to their perceived safety and the addition of flavorings extending the intended target market from adult cigarette smokers to include adolescents.^2^ This strategy has turned out to be treacherously efficient as 27.5% of US high schoolers in 2019 admitted to having vaped in the last 30 days.^3,4^ E-cigarettes are widely accepted to be a safer alternative to smoking,^4,5^ in particular causing less adverse effects on nonlung organs compared to traditional cigarettes.^4^ Still, alongside the surge in their use in the US and UK has come a rise in vaping-related lung injuries resulting in hospitalizations and deaths,^4,6,7^ with 2807 total hospitalized cases recorded by February 2020.^1^ Alarmingly, 78% of those admitted were under 35 years old, showcasing the danger facing the younger population.^8^ These patients were diagnosed with e-cigarette or vaping-related lung injury (EVALI), which involves diffuse alveolar damage.^9,10^ Most cases have been linked to a dilutant used in illicit tetrahydrocannabinol e-liquids, vitamin E acetate (VEA).^6^ This hydrophobic molecule was found to accumulate in the alveoli to cause lipoid pneumonia^11^ and to disrupt the pulmonary surfactant,^12−15^ hence resulting in widespread VEA bans.^16^ Despite this discovery, 20% of EVALI cases remain unexplained,^17^ and the respiratory health implications of many vaping components are still poorly understood.^4,18^

E-cigarette vapor is known to reach the alveoli, where any inhaled toxicants must first pass the delicate pulmonary surfactant film that sits atop the alveolar liquid. The surfactant has the vital biophysical function of reducing surface tension of the air–liquid interface in the alveoli.^19,20^ Without a functioning surfactant, high surface tension would prevent re-expansion after alveolar compression, resulting in alveolar collapse.^21,22^ Aberrant surfactant function and severe alveolar collapse are associated with acute respiratory distress syndrome (ARDS), which has been the final diagnosis of many advanced EVALI cases^6,14^ due to shared symptoms of alveolar damage and inflammation.^10,23,24^

A pulmonary surfactant is a membranous lipoprotein film synthesized by alveolar type II cells, which forms a monolayer with associated bilayers beneath in the aqueous subphase.^25^ It contains a mixture of lipids (90% of the total mass) and proteins (10% of the total mass). On the lipid side, 1,2-dipalmitoyl-*sn*-glycero-3-phosphocholine (DPPC) is the most essential component for the reduction of surface tension as it is the only lipid able to reach a compact gel-like liquid condensed state at physiological temperatures.^26,27^ Phospholipids with unsaturated acyl chains, such as 1-palmitoyl-2-oleoyl-*sn*-glycero-3-phospho-choline and -glycerol (POPC and POPG, respectively) prevent the interfacial monolayer from irreversible fracture at high compression levels by providing sufficient fluidity to the high content of saturated lipids, together with cholesterol.^28−30^ For additional film stabilization, the hydrophobic surfactant proteins SP-B and SP-C—alongside their roles in gas exchange^31^—work alongside unsaturated lipids to prevent film collapse,^32,33^ and thereby material loss. This is achieved by allowing the two-dimensional (2D) surfactant monolayer to fold into a three-dimensional (3D) structure at high compression levels through interlayer cross-links.^19^ Lipids with unsaturated acyl chains and proteins associate with the 3D buckled sublayer reservoir while phospholipids with saturated acyl chains—mainly DPPC—remain at the interface to self-assemble into tight lateral compaction yielding a near-gel structure, permitting the attainment of low surface tensions.^25,34,35^ The remaining hydrophilic surfactant proteins SP-A and SP-D are not surface-active; rather they play a role in the innate immune response.^36^

So far, studies on the effect of vaping on the biophysical properties of the pulmonary surfactant have had limited focus on specific components, and the conclusions have been inconsistent. A small number of studies report minimal to no surfactant disruption,^14,37,38^ whereas Graham *et al.* found significant increases in surface tension, *i.e.* surfactant disruption, postexposure to e-cigarette vapor.^39^ The biophysical impact on the surfactant of any specific vaping chemicals, therefore, remains essentially unknown. These chemicals include vaping flavorings, which are of particular importance as the main attraction for the younger demographic.^2,18^

Common vaping flavoring chemicals include aldehydes, such as the cherry flavoring benzaldehyde (BA), which is present in approximately 75% of vaping liquids.^40^ Aldehyde flavorings are widely used in the food and cosmetics industries; however, concerns have been raised for their impact on health in a vaping context, which has not yet been thoroughly explored.^4,40^ Recent studies found that vaping liquids containing aldehyde flavorings are chemically unstable,^41,42^ resulting in harmful byproducts not recorded in initial e-liquid product safety screenings.^5,18^ It was recently reported that during both storage and the heating process of vaporization, the base component propylene glycol (PG) and flavoring aldehydes react to form the respiratory irritant PG acetals, for example, benzaldehyde propylene glycol acetal (BPGA). It was observed that 40% of BA was converted to BPGA, with a carry-over rate of 50–80%.^41^

Considering BPGA is a highly hydrophobic molecule,^43^ in this study it was hypothesized that BPGA would sit at the air–liquid interface and interact with surfactant molecules to disrupt biophysical function. The effects of BA on the surfactant are also unknown; hence, it is being tested alongside BPGA. BA also serves as an additional control as a smaller and less hydrophobic molecule,^43^ thereby theoretically inducing a weaker effect.

Therefore, the aim of this study was to understand if and how the flavoring aldehyde BA and its byproduct BPGA disrupt the biophysical function of the pulmonary surfactant. To this end, the surface activity and their interactions with surfactant constituents were investigated for BA and BPGA, employing both a protein-free synthetic lipid surfactant (SLS)^44,45^ and a clinical surfactant containing surfactant proteins SP-B and SP-C (Alveofact).^46^ The SLS was developed after lipidomic analysis of human- and murine-derived surfactants^29,47^ and served as a control for vaping chemical–protein interactions.

Two different dynamic compression–expansion surfactometer models were utilized to investigate biophysical function: a quasi-static model (*i.e.*, the Langmuir–Blodgett trough, LBT, with compression–expansion cycles)^28^ and a model replicating physiological dynamics (*i.e.*, the constrained sessile drop, CSD).^48^ We monitored changes in the biophysical function of the surfactant formulations by quantifying three parameters, namely, the minimum surface tension, compressibility modulus, and hysteresis. To prevent alveolar collapse, a well-functioning human pulmonary surfactant brings the surface tension to below 2 mN/m, has a compressibility modulus that enables high compaction without film collapse, and demonstrates low hysteresis through compression–expansion cycles, defined as limited material loss and efficient lipid reorganization.^30,49,50^ Alterations to these three functional properties were observed in the presence of vaping chemicals, with a noteworthy implication of the hydrophobic surfactant proteins.

Molecular level insights into the interactions of BA and BPGA with surfactant lipids and proteins were obtained from atomistic molecular dynamics simulations. They revealed that both BA and BPGA partitioned to the surfactant monolayer and perturbed its structure. In simulations containing the surfactant proteins, the vaping chemicals accumulated in the vicinity of the hydrophobic proteins in the compressed monolayer, which explains the significant role of proteins observed in experiments.

Overall, we provide novel molecular insights into how a flavoring aldehyde inhaled from vaping chemicals and its *de novo* byproduct impacts surfactant function, which explains plausible toxicological mechanisms that would have implications on respiratory health.

## Results

### BA and BPGA Interfere with Lipids to Compromise the Biophysical Function of the Surfactant

**Figure 1:**
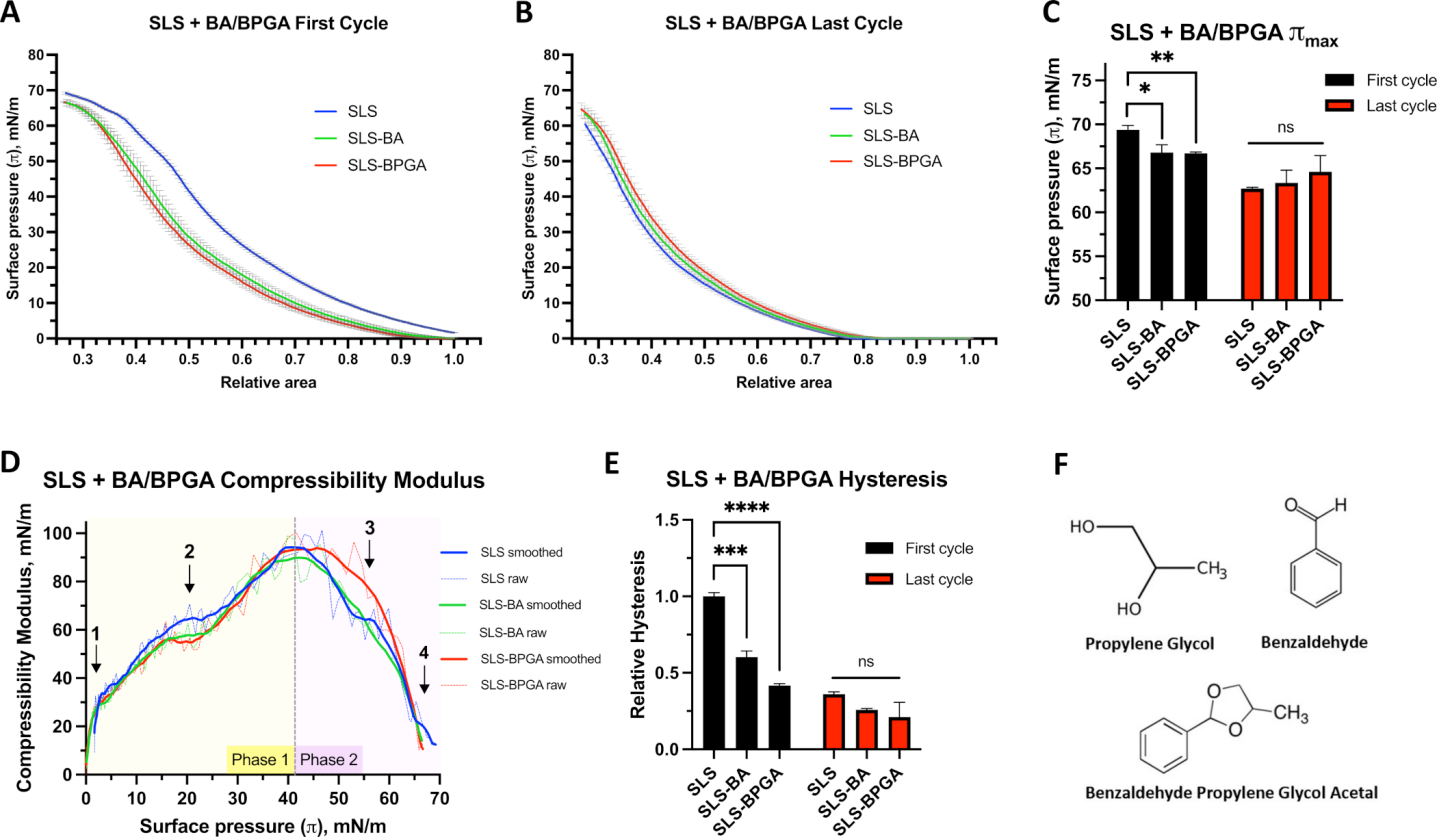
Molecular interaction study between SLS and BA or BPGA. Results from ten quasi-static compression–expansion LBT iso-cycles. (A) LBT Π–*A* isotherms from the first compression–expansion isocycle, three independent replicates. (B) Last cycle isotherm (out of a total of ten). SLS, SLS–BA and SLS–BPGA; three independent replicates. (C) Comparison of maximum surface pressures at the first and last (10th) cycles. (D) Compressibility modulus with both raw and smoothed data (Savitzky–Golay filter over nine points) shown. Arrows point to areas of interest in the graph (see text). (E) Hysteresis of the first and last cycles relative to the SLS first cycle. Experiments done at 25 °C. Preliminary results at 37 °C (Supporting Information Figure S2) did not allow us to investigate molecular interactions at high surface pressures. (F) Investigated vaping compounds. Significance values represent results from two-Way ANOVAs; “ns”: not significant (*p* > 0.05), *p < 0.05, **p < 0.01, ***p < 0.001, ****p < 0.0001.

It is known that changes in the lipid composition or variations from the evolutionary refined ratio of saturated and unsaturated chains of lipids and cholesterol drastically influence the biophysical function of lung surfactant monolayers,^29,32^ multilayered films, and the dynamics and interconnections between the two.^25,33^ To determine the molecular interactions between components of the SLS—which consists of the major surfactant lipids—and the vaping components BA and BPGA, we quantified its biophysical function by means of monitoring the surface pressure at the air–liquid interface on an LBT. We performed 10 consecutive compression–expansion cycles (Π–*A* iso-cycles), as monolayer constituent refinement is known to be an important property of a lung surfactant^20^ (Supporting Information Figure S1). These were performed with quasi-static LBT compression rates to allow the observation of fine molecular interactions.^51,52^ In the Π–*A* isotherm measured during the first cycle, there was a clear decrease in the Π_max_ when either BA or BPGA was added (Figure 1A). Therefore, the interaction of BA and BPGA with surfactant lipids prevents the achievement of higher surface pressures. Another interesting aspect to note was that below 10 mN/m—where a liquid expanded (L_e_)-like phase could be expected—neither BA nor BPGA seemed to perturb the surface pressure. This is evident from the similar slopes of increase in the surface pressure measured in the absence or presence of either BA or BPGA. However, once the liquid condensed (L_c_)-like phase is reached at surface pressures ≈50 mN/m or above—where a possible multilayered material could be associated with the interfacial monolayer—vaping components diminished the effect as can be observed by a smoothing of the kinks between 48 and 63 mN/m. Notably, this effect was less prominent in the last cycle isotherms (Figure 1B). After a cycling process and refinement of the monolayer, the Π_max_ was significantly reduced and the kinks also vanished (Figure 1B,C).

Ideally, the compressibility modulus (κ) is high, leading up to maximum surface pressures as tight lateral compaction is essential to reach Π_max_. Conversely, lower κ is favorable at high surface pressures as film elasticity prevents irreversible collapse—and thereby material loss—by better enabling 2D to 3D transitions. The right balance between fluid and gel phases at a high surface pressure is an essential feature to allow buckling of the monolayer to the subphase in a reversible manner; a pure DPPC monolayer allows reaching the highest surface pressure, but it fractures.^28,29^ In a protein-free model, mainly unsaturated lipids would move into the 3D reservoir at high surface pressures, especially beyond the “squeeze-out” plateau.^53^ The compressibility modulus was calculated throughout the first cycle exclusively as this is where it is best defined.^49,54^ We observed a biphasic behavior with alterations to SLS compressibility throughout both phases seen with BA and BPGA addition (Figure 1D). During the incline toward the maximum compressibility modulus (phase 1 in Figure 1D), κ was smaller at smaller surface pressures with BA or BPGA present as compared with SLS alone (point 1 in Figure 1D). At a surface pressure of 20 mN/m (point 2), BA and BPGA reduce SLS κ from 69 to 58 and 56 mN/m, respectively. In the decline (phase 2), SLS–BPGA displays a slower decrease in compressibility modulus than SLS and SLS–BA, increasing κ by over 10 mN/m at Π = 55 mN/m (point 3). At a surface pressure larger than 65 mN/m (point 4), both vaping chemicals present loss of the characteristic SLS “kink”, depicting a solid gel phase of lipid compaction, which enables SLS to reach 70 mN/m Π_max_. Overall, BA and BPGA negatively influence the surfactant lipid compressibility modulus κ at high and low surface pressures.

To sustain high surface pressures over multiple cycles, the loss of surfactant constituents from the air–liquid interface at high surface pressures must be avoided, while allowing monolayer refinement to optimize film organization.^30^ Low hysteresis reflects minimal loss of surface-active material between compression and expansion. That said, BA and BPGA both significantly decrease first cycle SLS hysteresis (Figure 1E). We observed a 40 and 60% decrease with BA and BPGA, respectively, with both *p*-values below 0.001. After ten iso-cycles, hysteresis had decreased by over 60% in all groups, indicating refinement. Notably, variation between groups was also drastically reduced, with the SLS–BA *p*-value increasing to above 0.05. As such, BA and BPGA interfere with the initial surfactant lipid hysteresis. In summary, the three biophysical function parameters confirm fine interactions between surfactant lipids and BA and BPGA molecules; however, these seemed unstable over multiple quasi-static iso-cycles.

### BPGA Partitions to the Acyl Chain Region of SLS Monolayers and Increases Their Packing

**Figure 2:**
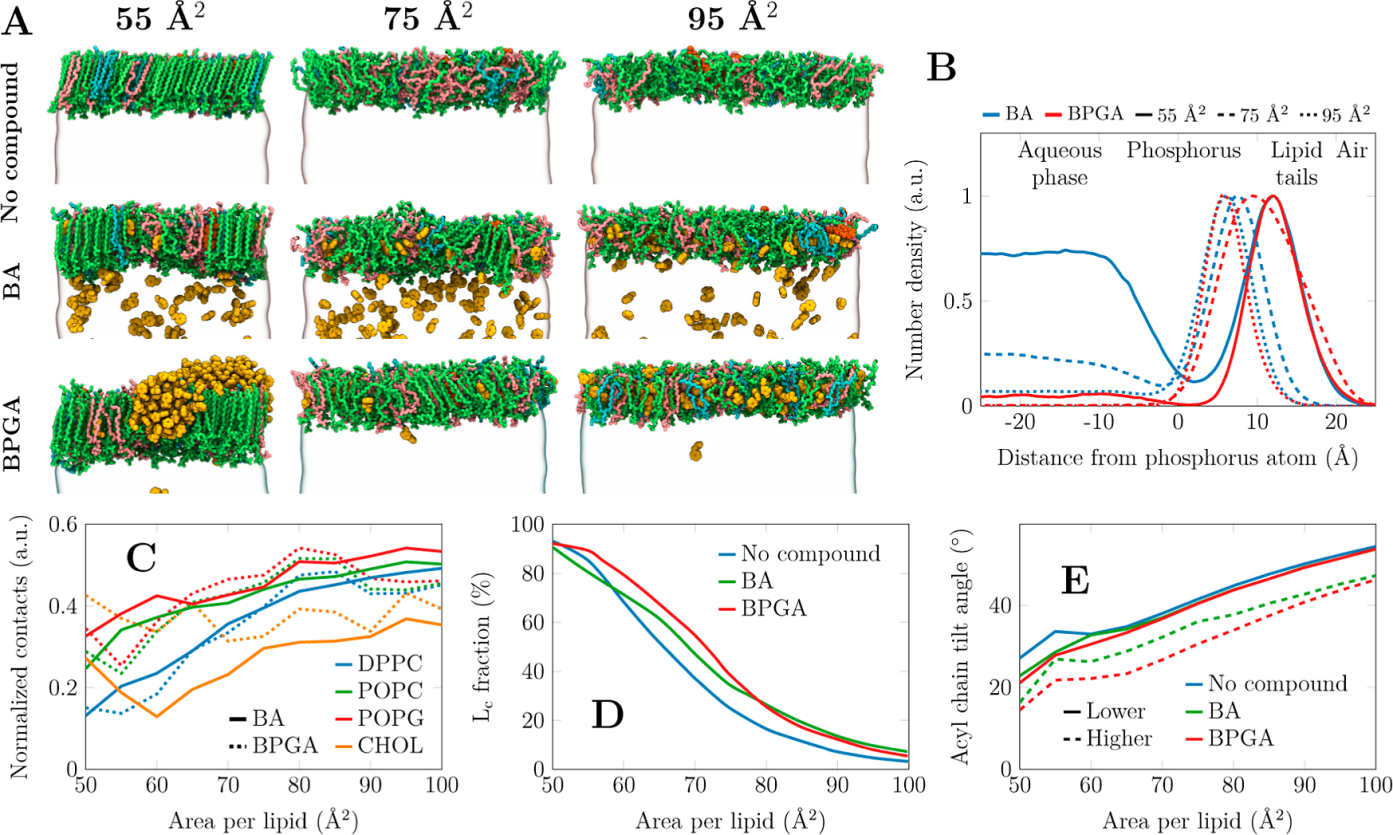
Partition preferences of the vaping chemicals into the SLS monolayer from atomistic molecular dynamics simulations. (A) SLS monolayers composed of 149 lipids per monolayer, containing no vaping chemicals (top row), 320 benzaldehyde (BA) molecules (middle row), or 200 benzaldehyde propylene glycol acetal (BPGA) molecules (bottom row). The partitioning tendency at 10-fold smaller compound concentrations was similar (Figure S6), yet there were not enough vaping chemical molecules for aggregation. The final structures of simulations at three areas per lipid are shown. DPPC, POPC, POPG, and cholesterol are depicted in green, pink, cyan, and orange, respectively, whereas the vaping compounds are shown in yellow. Water is shown as a transparent surface, and all hydrogens are omitted for clarity. (B) The density profiles of BA and BPGA were across the lipid monolayer at the air–water interface. Data are shown at three compression states also visualized on the left side of the figure. The density profiles are normalized so that their maxima are set to 1. (C) Interaction preference of BA and BPGA with different lipids. As the lipid moieties are present in different amounts, the contacts are normalized by the number of possible interactions. (D) Fraction of acyl chains and cholesterol molecules that are tightly packed, hence resembling the L_c_ phase. (E) The tilt angle of the phospholipid acyl chains was obtained with no vaping chemicals as well as with lower and higher concentrations of either BA or BPGA.

Having observed that BA and BPGA interactions with surfactant lipids at continuous quasi-static compression detrimentally influence the functional surface pressure of SLS monolayers, we set out to resolve the possible molecular mechanisms that would lead to this behavior. An obvious element of differential behavior between BA and BPGA would be their different hydrophobic/hydrophilic moieties. Our results indicated that vaping interactions vary depending on the surface pressure, which suggests the role of the lateral packing properties. To investigate this at the molecular level, we performed all-atom molecular dynamics simulations using our well-validated simulation approach that captures the physics of the air–water interface.^44,45,55,56^ We simulated the SLS composition of monolayers with two concentrations of BA and BPGA and across a range of compression states. Figure 2A shows snapshots of the SLS monolayers containing a higher concentration of BA and BPGA at three selected area per lipid (APL, Å^2^) values. APL reflects different surface pressures; *i.e.* the lower the APL, the higher the surface pressure. These snapshots determine the preferential partition properties of the vaping chemicals in an SLS monolayer. Overall, BPGA shows a high partitioning preference to the nonpolar lipid acyl chain region at all APL values, whereas some BA always remains in the aqueous phase. Curiously, at higher concentrations, BPGA is not very soluble in the lipid phase at small APL (55 Å^2^) and forms aggregates at the lipid–air interface. Still, in a 10-fold lower concentration, BPGA is readily soluble also in the compressed monolayers, while some BA remains in the aqueous phase (Figure S6).

The partitioning of vaping chemicals is quantified by density profiles in Figure 2B. Here, the normalized (maximum = 1) number density across the monolayer normal is shown. The curves confirm the visual observation from Figure 2A; BPGA always partitions to the lipid phase, whereas a substantial fraction of BA remains in the aqueous phase. In the compressed monolayer with an APL of 55 Å^2^, BPGA resides in the acyl chain region and is depleted from the polar headgroup region, whereas BA has significant populations in the acyl chain region, in water, and also in the headgroup region. At an intermediate APL of 75 Å^2^, BA prefers the polar interface and water, whereas BPGA resides deeper in the monolayer. In the very loosely packed monolayer at 95 Å^2^, both chemicals reside in the headgroup region of the thin monolayer.

Next, we looked into the selective interactions of BA and BPGA with different lipid species by analyzing the vaping chemical–lipid contacts from the simulations. As demonstrated in Figure 2C, both vaping compounds show no specificity toward any lipid type at large APLs, *i.e.* in the loosely packed monolayer. However, upon compression, both BA and BPGA demonstrate more interactions with the lipids that have unsaturated acyl chains, namely, POPC and POPG in the SLS mixture. This result suggests that the compounds are excluded from the tightly packed DPPC acyl chains and do not significantly perturb their adaptation into the L_c_-like phase. This presence of BA and more so of BPGA in the monolayer and especially among the unsaturated acyl chains naturally has implications for its structure. Cholesterol shows very different trends for the two compounds: it shows little interaction with BA at all compression states and with BPGA at a large APL. However, at a low APL, BPGA significantly interacts with cholesterol. Our visual analysis suggests that the BPGA clusters in the monolayer (Figure 2A) gather hydrophobic cholesterol molecules around them, thus seemingly depleting the remainder of the monolayer of cholesterol.

MD simulations also suggest that the presence of BA and BPGA leads to a tighter monolayer packing. The chemicals residing among the unsaturated acyl chains at a constant area lead to a larger amount of lipid chains being assigned with the L_c_-like packing. This effect is demonstrated in Figure 2D. At large APLs, essentially no chains are packed, and the chemicals have little effect. At small APLs, on the other hand, essentially all acyl chains are packed tightly despite the presence or absence of BA or BPGA. At intermediate areas, on the other hand, both BA and BPGA promote lipid packing, and in the range from 55 to 75 Å^2^, the effect of BPGA is more significant.

One notable effect of BA and BPGA at high compression is that they decrease the average lipid tilt. As demonstrated in Figure 2E, in the compressed monolayer and in the absence of vaping chemicals, lipid acyl chains adapt a conformation with a typical L_c_-like tilt of ≈25° observed in experiments^57^ as well as in previous simulations of compressed lipid monolayers.^44,56^ However, even when a small amount of BA or BPGA is added, the tilt angle in the compressed monolayer decreases by ≈5°. With a larger concentration of vaping chemicals interacting with the monolayer, the acyl chain tilt angles are decreased across all compression states by ≈10°.

### BA and BPGA Interactions with Surfactant Lipids under Physiological Dynamics Do Not Significantly Influence the Biophysical Function

**Figure 3:**
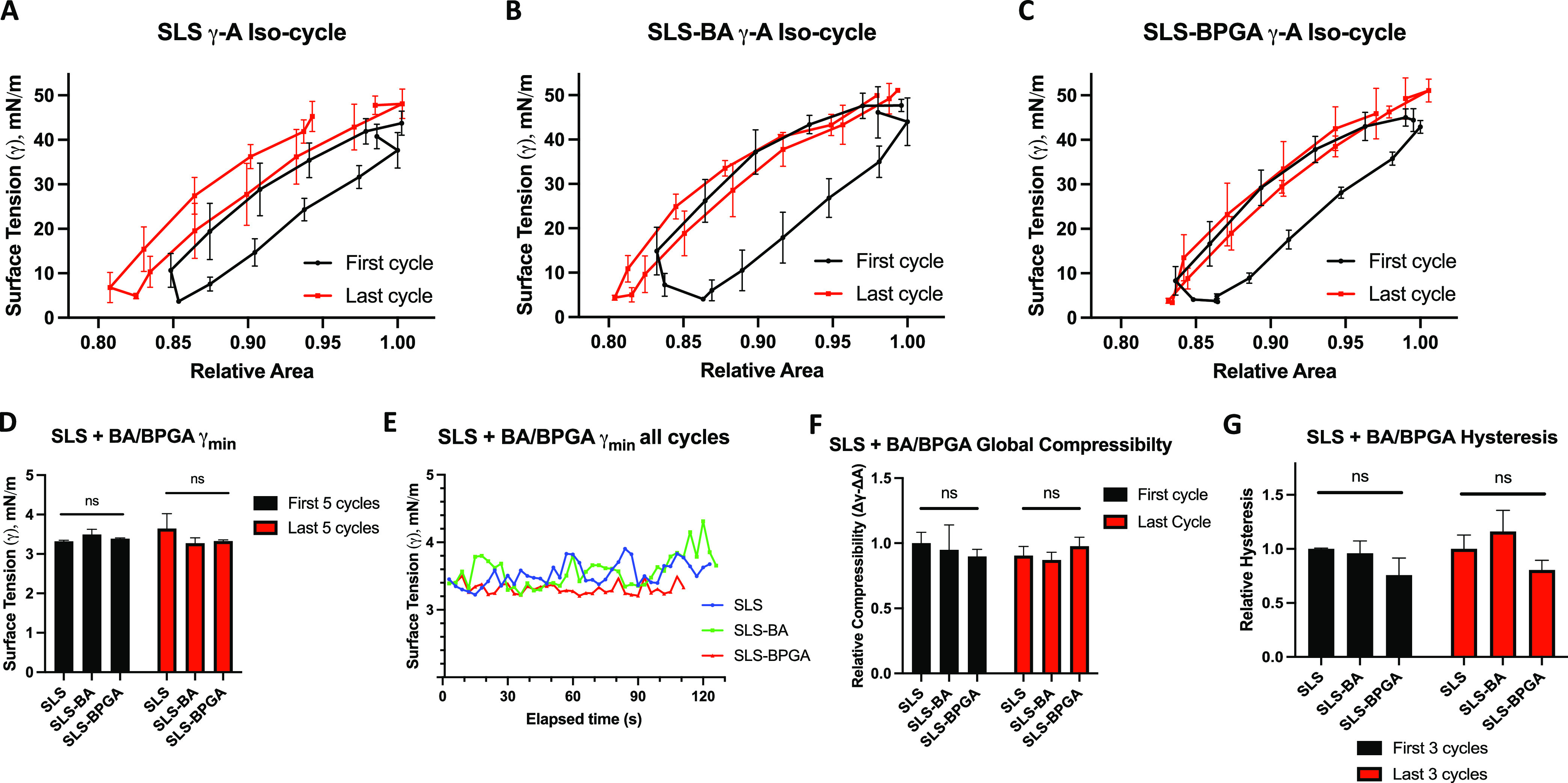
Physiological compression–expansion cycles for SLS and vaping components. Results from CSD cycles run at up to 20 cycles per minute for 2 min. (A) SLS alone, first and last iso-cycles. (B) SLS with BA, first and last iso-cycles. (C) SLS with BPGA, first and last iso-cycles. BA and BPGA were added in a 1:10 molar ratio to surfactant lipids. (D) Comparison of the mean γ_min_ of the first and last five cycles for each condition. (E) Temporal evolution of γ_min_ covering all cycles of each condition represented. (F) Relative global compressibility of the first and last cycles, relative to the SLS first cycle. (G) Mean hysteresis of the first and last three cycles, relative to the SLS first cycle. Three independent replicates for each condition. Significance values represent results from a two-way ANOVA; “ns”: not significant (*p* > 0.05).

To confirm the physiological relevancy of interactions seen with SLS on the LBT, the effect of BA and BPGA were tested in physiological compression–expansion cycles on the CSD surfactometer. As seen in Figure 3A–C, minimal differences were observed between the shapes of the γ–*A* iso-cycles of each condition. This could be confirmed after observing the lack of significance between groups for all three functional parameters (γ_min_, global compressibility, and hysteresis) during both first and last cycles (Figure 3D–G). Overall, the minimal loss of BA and BPGA surface-active properties under physiological dynamics indicates little interaction between SLS and BA or BPGA. This was a rather surprising result, and we thus proceeded to validate it by assessing the interfacial behavior of BA and BPGA in physiological compression–expansions in a dose-dependent manner. Each chemical’s surface tension was measured on the CSD in increasing concentrations (Supporting Information Figure S3). 1 mg/mL BPGA had higher surface activity than BA at the same concentration, reducing the surface tension from a mean of 72 to 48 mN/m rather than 67 mN/m as seen with BA. Increasing concentrations of both chemicals consistently decreased surface tension, apart from 1 g/mL BPGA, which sank immediately due to high density. Considering the statistical analysis completed (Figure 3D,F,G), these results imply that BA and BPGA remain surface-active at the air–liquid interface over multiple cycles and while at the interface, they interfere minimally with the surface tension maintained by SLS.

### BA and BPGA Alter the Biophysical Function of Alveofact at Physiological Compression–Expansion Rates

Our previous results indicate that physiological dynamics induce loss of lipid interactions with BA and BPGA. Still to be investigated is whether the addition of surface-active surfactant proteins would influence the chemical interactions of vaping components at the interface and whether this would disrupt the biophysical function of the surfactant.

Alveofact is a commercial bovine-derived clinical surfactant containing the hydrophobic surfactant proteins SP-B and SP-C.^46^ In attempts to assess whether the addition of SP-B and SP-C would result in additional molecular interactions with BA and BPGA from those observed with surfactant lipids, Alveofact was tested on the LBT. In contrast to SLS (Supporting Information Figure S4A), Alveofact would not reach the standard Π_max_ of >70 mN/m,^36^ rather plateauing at 50 mN/m. This limit was confirmed by increasing the molar concentration of Alveofact up to 5-fold (Supporting Information Figure S4B). Therefore, LBT measurements would not be informative of changes to biophysical properties at physiologically relevant high surface pressures.

**Figure 4:**
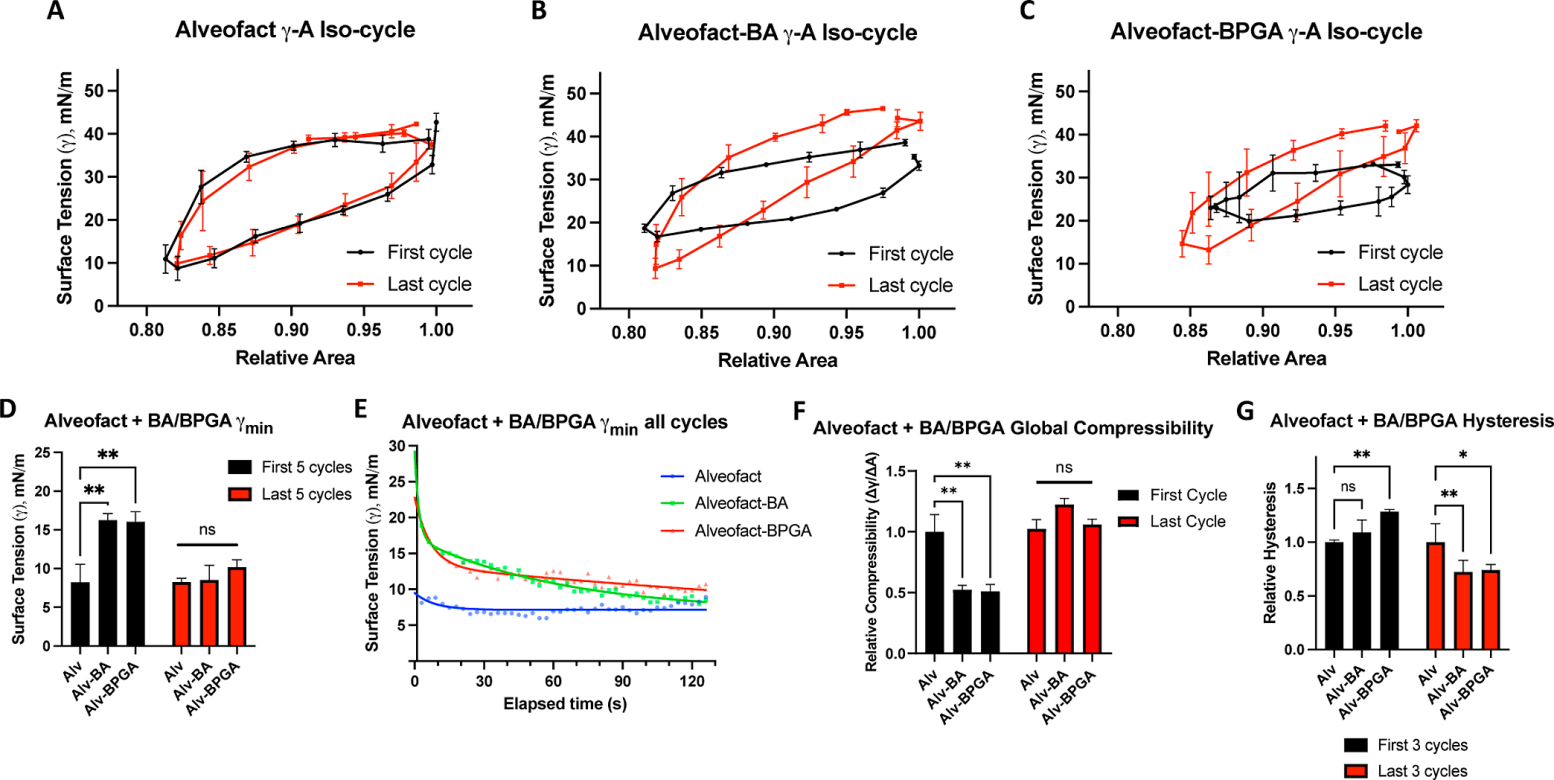
Physiological compression–expansion cycles for Alveofact and vaping chemicals. Results from CSD cycles run at up to 20 cycles per minute for 2 min. (A) Alveofact alone, first and last γ–*A* iso-cycles. (B) Alveofact with BA, first and last γ–*A* iso-cycles. (C) Alveofact with BPGA, first and last γ–*A* iso-cycles. BA and BPGA were added in a 1:10 molar ratio to the major surfactant lipids. (D) Comparison of mean γ_min_ of the first and last 5 cycles for each condition. (E) Temporal evolution of γ_min_ over all cycles for each condition. Alveofact alone is fitted to a monoexponential decay curve. Alveofact–BA and Alveofact–BPGA are fitted to biexponential decay curves. (F) Global compressibility of the first and last cycles relative to the first cycle of Alveofact. (G) Mean hysteresis of the first three and last three cycles, relative to the first three cycles for Alveofact. Three independent replicates for each condition. Significance values represent results from a two-way ANOVA; “ns”: not significant (*p* > 0.05), *p < 0.05, **p < 0.01.

For this reason, Alveofact experiments progressed to the CSD, where it was theorized that fast physiological compression–expansion rates of up to 20 cycles per minute^50^ would force rapid lateral self-assembly, reducing the accumulation of surfactant constituents into the 3D reservoir and thus enabling the achievement of γ_min_. This would allow the observation of physiologically relevant molecular interactions. To this end, the effects of vaping chemicals on the biophysical function of Alveofact were evaluated over 40 physiological compression–expansion cycles (Supporting Information Figure S5). BA and BPGA caused visible alterations to Alveofact γ–*A* iso-cycles extracted from cycle data (Figure 4A–C). While Alveofact iso-cycles remain stable throughout multiple cycles, BA and BPGA addition seem to induce iso-cycle deformation, especially during the first iso-cycle. Interestingly, these observations exhibit interference of surface tension and area change and, thereby, biophysical properties.

To quantify iso-cycle alterations, first, γ_min_ were extracted (Figure 4D,E). In the first cycles, there were significant increases in the mean surface tension from 8 mN/m with Alveofact alone to 16 mN/m with either BA or BPGA included (Figure 4D). This effect was lost during the last five cycles, during which BA- and BPGA-containing mixtures have similar γ_min_ values to Alveofact alone. This trend was reflected in exponential decay curves representing the kinetics of all cycle γ_min_ values (Figure 4E). A shorter decay half-life indicates faster reduction in γ_min_, *i.e.*, an improvement in the biophysical function. While Alveofact alone fits a monoexponential curve and quickly plateaus to a constant γ_min_ of ≈7 mN/m, indicating rapid monophasic improvement, the addition of BA or BPGA causes a shift to biexponential curves, which plateau at higher surface tensions. This infers that the rapid decrease is coupled with a long-term interference of the biophysical function, with BPGA inducing the most persisting disruption to γ_min_ with a second phase half-life five times slower than that of BA (253.2 s compared to 44.52 s). In brief, the experiments suggest that BA and BPGA interfere with the biophysical function of the surfactant by interacting with the surfactant proteins SP-B and SP-C in physiological compression expansions over time scales of minutes.

To assess the effects of protein–vaping chemical interactions on film compaction and elasticity, the global compressibility modulus was calculated *via* the iso-cycle slope.^49^ BA and BPGA significantly reduce the global compressibility modulus κ of Alveofact during the first cycle by ≈50%, although this is restored during the last cycles (Figure 4F). Therefore, protein–vaping chemical interactions initially decrease the lateral film compaction, although this effect is not sustained under physiological dynamics. Finally, to determine if BA and BPGA induce alterations in membrane organization over multiple physiological compression–expansion cycles, Alveofact hysteresis was extracted from the first and last γ–*A* iso-cycles. Hysteresis of the first five cycles increased with BA and BPGA addition, although BPGA imposed the only significant change (Figure 4G). Interestingly, during the last 5 cycles, BA and BPGA both significantly *decrease* Alveofact hysteresis. Protein–vaping chemical interactions thereby induce persistent changes to film organization. BA and BPGA induce material loss in the first cycles, which, in turn, presents a more refined—yet possibly less functional—monolayer by the last cycles compared to Alveofact alone.

### BA and BPGA Interact with Surfactant Proteins in the SLS Monolayer

**Figure 5:**
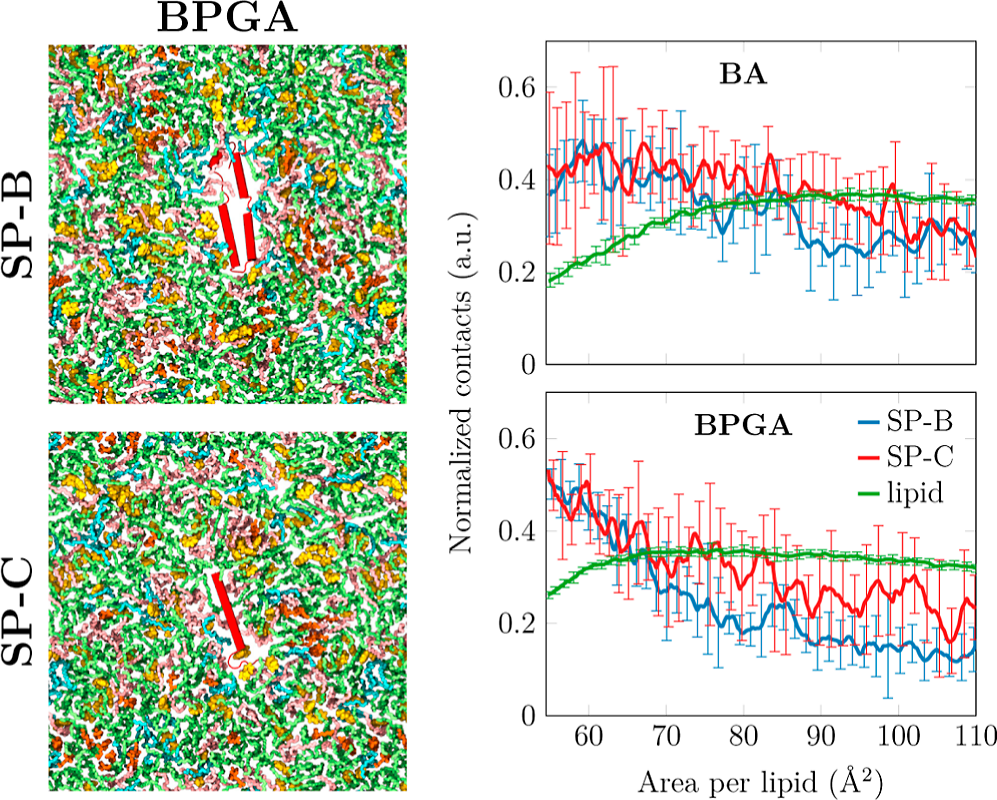
Interaction of vaping chemicals with hydrophobic surfactant proteins SP-B and SP-C. Left: Snapshots of the simulation system containing one copy of either SP-B (top) or SP-C (bottom). The simulations shown also contain BPGA, shown in yellow. Proteins are colored red, DPPC green, POPC pink, POPG blue, and cholesterol in orange. Hydrogens and water are omitted for clarity. Right: Interactions of BA and BPGA with the surfactant lipids and proteins as a function of the monolayer area. The contact numbers are normalized by the number of possible interactions in the two groups included in the analyses. For lipids, only the curve from the simulation containing SP-B is shown since the result with SP-C is essentially identical. The error bars show the difference between the data calculated from the two replica simulations.

Our experiments on Alveofact suggested that BA and BPGA could interact with the surfactant proteins SP-B and SP-C, leading to persistent negative impacts on the parameters characterizing the biophysical function of the surfactant—namely, γ_min_ and hysteresis under physiological compression–expansion dynamics. To validate this hypothesis, we performed additional atomistic molecular dynamics simulations of surfactant monolayers containing either SP-B or SP-C. Moreover, these simulations followed the experiments, as BA or BPGA was first allowed to interact with the monolayer, after which it was compressed from a large area per lipid of 110 Å^2^ (Π ≈ 0 mN/m) to a small one of 55 Å^2^ (Π ≈ 70 mN/m) during the course of a 2 μs-long simulation. Examples of the simulation systems with BPGA are shown in the left panel of Figure 5.

We calculated the number of contacts between the vaping chemicals and the lipids or the protein as these values characterize their preferential interaction partners at different compression states. As demonstrated on the right panel of Figure 5, the interactions with lipids are somewhat sensitive to the compression state. At low areas per lipid, more BA remains in the aqueous phase, leading to a steady decrease of contacts below ≈80 Å^2^. The same exclusion takes place with BPGA, but to a smaller extent due to its larger partitioning preference toward the hydrophobic acyl chain region (Figure 2A,B).

Surprisingly, the interaction of BA and BPGA with the surfactant proteins demonstrates a different trend. When the monolayer is compressed, both BA and BPGA accumulate near the proteins. In this state, interactions with SP-B and SP-C are equally likely. However, these contact numbers are normalized by the possible interaction partners, signaling that the larger SP-B interacts overall with more vaping chemicals. BPGA demonstrates more interactions with the proteins in the compressed monolayer. This is also visualized in Figure S8, which shows the accumulation of BPGA onto the SP-B surface as a function of compression. Curiously, we could not identify any specific binding modes or differentiate between the benzene or glycol acetal parts of BPGA as favorable interaction partners with SP-B. Instead, the overall hydrophobic BPGA and BA seem to accumulate on hydrophobic regions of SP-B. Altogether, our atomistic simulations confirm that both BA and BPGA indeed interact with the surfactant proteins especially in the physiologically relevant compressed surfactant, leading to the compromised biophysical function of Alveofact observed in the experiments.

## Discussion

In this study, we evaluated the effects of the highly hydrophobic e-liquid byproduct, BPGA, and its hydrophobic precursor, the flavoring aldehyde BA, on the biophysical function of surfactant under dynamic conditions. Our aim was to assess the role of common vaping flavorings in surfactant dysfunction. It was hypothesized that these components may affect the surfactant due to their hydrophobic nature, thereby enabling interactions with surfactant molecules at the air–liquid interface. BPGA, as the more hydrophobic molecule, was expected to have a stronger impact on surfactant biophysical function than BA.^43^

Fine interactions between surfactant lipids with both BA and BPGA were confirmed at quasi-static compression–expansion rates by employing a lipid-only SLS model (Figure 1). These interactions were further explained in our atomistic molecular dynamics simulations, which demonstrated the partitioning of both BA and BPGA into the SLS monolayer at all compression states, with BPGA exclusively residing in the acyl chain region at high surface pressures. As hypothesized, the hydrophobicity of these molecules is very likely the enabler of these interactions.

The fine interactions observed in the quasi-static model resulted in BA and BPGA significantly reducing the initial maximum surface pressures (Figure 1A–C). This is likely explained by the additional negative impact seen on compressibility (Figure 1D), as both chemicals impaired lateral compaction at low surface pressures and maximum surface pressure, where it was observed that lipids did not self-assemble into the gel phase. Furthermore, the initial SLS hysteresis was significantly reduced (Figure 1E). While this does not directly indicate loss of surface-active material, it may imply a lack of film refinement, preventing optimization of monolayer arrangement.^30^ The effect of BA and BPGA observed on the compressibility modulus at a surface pressure of ≈20 mN/m (Figure 1D) coincides with the region where ordered L_c_-like domains start to form in the surfactant.^44^ With BA or BPGA present in the membrane (Figure 2C), a decrease in the overall lipid packing is notable and consequently is translated to the compressibility modulus. At high pressures, the compressibility modulus is increased by the presence of BPGA (Figure 1D). This coincides with the aggregation of BPGA molecules in the monolayer (Figure 2A) at this pressure range, and these clusters could resist compression and temporally stabilize the interface during compression–expansion cycles as observed by the prominent kinks close to the equilibrium surface pressure during the expansion process in the LBT isotherms (Figure S1C). We interpret that this effect is due to BPGA being more hydrophobic and “bulkier” than BA as per our hypothesis.

Notably, the significant effects by BA and BPGA on Π_max_ and hysteresis were either lost or considerably reduced over the 10 quasi-static compression–expansion cycles with SLS (Figure 1B,C,E). A potential explanation may be the “squeeze-out” hypothesis, where the surfactant lipids with saturated acyl chains, *i.e.*, DPPC, force other molecules out from the monolayer at high compressions.^58^ This would usually cause material rearrangement into the 3D structure reservoir; however, in a lipid-only model, the lack of proteins limits the stability of unsaturated phospholipid sublayer formation, which increases collapse and therefore loss of chemicals directly into the subphase. An effective lung surfactant displays a perfect balance between being stiff enough to reach and sustain high surface pressures recurrently over the breathing cycles and the flexibility to generate buckled areas attached beneath the interfacial monolayer, hence avoiding collapse or irreversible loss of material. As mentioned above, seemingly even in the absence of surfactant proteins, BPGA could stabilize temporarily high surface pressure likely by decreasing the formation of irreversible fractures during expansion. Unfortunately, the sizes of atomistic simulation systems are too small to observe this “squeeze-out”. Still, the decrease in surface pressure during the first cycle could well result in the excessive structural perturbation summarized in Figure 2. Here, the vaping chemicals interact preferably with the unsaturated lipid chains and thus affect the phase behavior of the surfactant, including the decrease in the L_c_ phase lipid tilt characteristic required for reaching the physiological Π_max_.

Unexpectedly, the fine surfactant lipid–chemical interactions were then proven insignificant under physiological compression–expansion dynamics, as SLS CSD results presented no significant differences in any of the three functional parameters (Figure 3). Therefore, BA and BPGA are likely not able to significantly disrupt the pulmonary surfactant biophysical function *via* lipid interactions. A probable reason is immediate chemical loss from the interface imposed by rapid compression rates, forcing abrupt maximum lateral compaction, after it was observed that BA and BPGA would normally impose surface tension reduction under these conditions (Supporting Information Figure S3). Fast lateral compression has been observed to eventually force components out of the monolayer toward the linked bilayers beneath,^32,44,53^ which seems to be a very plausible mechanism with vaping components too.

Once the fine lipid–chemical interactions initially observed seemed to have physiological insignificance, the investigation turned to a new hypothesis, potential interactions between BA and BPGA and the surface-active surfactant proteins SP-B and SP-C. To this end, we employed the clinical surfactant Alveofact.^46^ Unfortunately, attempts at fine interaction studies with Alveofact at quasi-static rates (Supporting Information Figure S4) were identified as abnormal due to the knowledge that Alveofact must reach γ_min_ below 2 mN/m to be an effective surfactant substitute in premature neonates.^20,46^ A similar behavior of Alveofact has also been previously reported on the LBT.^59^ The slow quasi-static compression rates likely enable SP-B and SP-C to facilitate extensive monolayer folding, or 2D to 3D transition, reducing maximum lateral lipid compaction by Π_max_.^25,34^

Physiological compression–expansion rates provided a solution by forcing rapid lateral compaction to overcome protein-facilitated film elasticity. Under physiological dynamics, BA and BPGA were proven to induce persistent disruption of the biophysical function of Alveofact *via* stable vaping chemical–protein interactions after long-term interference of both γ_min_ and hysteresis were observed (Figure 4E,G). Essential protein involvement was deduced as protein content is the most prominent difference between SLS and Alveofact, and therefore, the most plausible reason for differences observed at physiological dynamics. That said, SLS and Alveofact do differ in lipid composition.^60^ Although the ratio between saturation and unsaturation was kept within a similar range, in particular, cholesterol concentration variation will have an impact on the membrane fluidity and hence in the viscoelastic properties of the surfactant. Thereby, it affects the formation of 3D structures at high compression, although, notably, we did not observe cholesterol to significantly participate in direct interactions with the vaping chemicals (Figure 2C). The difference in structures resulting from the difference in lipid composition, along with the addition of hydrophobic surfactant proteins, changes the way BA and BPGA interact with the surfactant films and the related physical and chemical behavior. Therefore, the validation of protein involvement was indeed necessary in our atomistic molecular dynamics simulations.

Surfactant protein–vaping chemical interactions were observed in atomistic molecular dynamics simulations of protein-containing monolayers under dynamic compression. Whereas compression leads, on average, to fewer lipid–vaping chemical interactions, the number of surfactant protein–vaping chemical interactions actually increased (Figure 5). This unexpected behavior is explained by the shift of SP-B and SP-C away from the lipid–liquid interface^45^ upon compression. Together with the surrounding lipid acyl chains, the proteins thus form a hydrophobic moiety, which the hydrophobic chemicals occupy rather than remain among the tightly packed acyl chains. These excessive interactions suggest that BA and especially BPGA can perturb the functions of the proteins in this physiologically relevant low surface tension state. These data support our hypothesis that these vaping chemicals can disrupt the surfactant biophysical function, although specifically through hydrophobic surfactant proteins, which was not previously foreseen. BA and BPGA, therefore, have the potential to interfere with the maintenance of low surface tensions in the alveoli when inhaled, thereby risking alveolar collapse, which would lead to respiratory damage and distress.

Vaping chemical interactions with SP-B and SP-C would also provide reasoning for the near-immediate decrease of effect on all biophysical function parameters (Figure 4), which is especially evident in the rapid first phase of the γ_min_ decrease (Figure 4E). This first phase with higher biophysical impact may represent preoptimization of film arrangement, where BA and BPGA have not yet been fully associated with the surfactant proteins, at which point they would be able to transiently enter the 3D reservoir along with SP-B and SP-C throughout multiple maximum compressions.^25,34^ This may avoid permanent “squeeze-out” from the surfactant film while also preventing chemicals interfering with surface-active surfactant lipid self-assembly at points of high lateral compaction. Thereby, the protein–chemical interactions help retain the chemicals in the surfactant, leading to some long-lasting interference to the surfactant’s biophysical function, as evidenced in Figure 4E. However, it is likely that much of the vaping chemical is still being lost to the subphase from the 3D reservoir, as there remains a downward tendency in γ_min_ after the initial first phase of decline (Figure 4E). In a cellular model, this may have negative implications, as once past the surfactant film, chemicals can freely diffuse through the alveolar liquid to reach the alveolar–capillary barrier, where they may induce cytotoxicity.^61^ An inflamed alveolar epithelium can in turn lead to further surfactant disruption *via* exposure to foreign molecules.^62^ It is then pertinent that the cytotoxicity of these chemicals to the alveolar epithelium should be studied in future work, as damage to the alveolar–capillary barrier would have significant health implications. This is a novel second potential mechanism of respiratory toxicology by these vaping chemicals revealed in this study.

Throughout the study, it was observed that BPGA had a similar but greater effect than BA on the surfactant biophysical function. BPGA was shown to induce more durable and stronger interactions with surfactant lipids and proteins in the different models. This can be explained by greater surface activity (Supporting Information Figure S3) and greater aggregation in the monolayer at high compressions (Figure 2A), which is visible in the long expansion plateau right after reaching the highest surface pressure (Figure S1C), as well as higher hydrophobicity. The proposal that BA alone has the potential to disrupt surfactant function and therefore possibly lead to lung damage is concerning. BA is a highly common vaping flavoring, present in approximately 75%^40^ of flavored e-liquids popular in the younger population. Furthermore, BA is far from the only flavoring aldehyde used in e-liquids, which all have acetal byproducts. Others commonly used include vanillin and cinnamaldehyde.^63^ In the future, a wider range of aldehyde flavorings should be tested to assess whether all of these chemicals have similar effects on the biophysical activity of the surfactant, thereby helping to understand the extent of the issue.

## Implications

This study is the first to propose and validate if and which specific vaping liquid chemicals can react with lung surfactant monolayers. Findings reveal that flavoring aldehyde BA and its byproduct, BPGA, interact with surfactant monolayers and can significantly disrupt surfactant biophysical properties *via* interactions with surfactant proteins SP-B and SP-C. This is a respiratory toxicology mechanism for alveolar collapse and therefore has significant implications for human respiratory health. We also provide evidence to suggest that upon continuous inhalation, vaping chemicals will be dragging surfactant components down to the aqueous subphase. In this case, there is a high likelihood that these chemicals would reach the following layer of the alveolar–capillary barrier, *i.e.*, the alveolar epithelium, where they may induce cytotoxicity. This highlights the widely unconsidered potential dangers of including food-grade flavorings, such as the commonly used aldehyde chemicals, in vaping products or products for inhalation. Importantly, this study also emphasizes the need to investigate the health implications of *de novo* vaping byproducts, not only the stated ingredients. We strongly advise that this research is taken into account by regulatory bodies in regards to reassessing if food-grade flavorings are safe-to-inhale and considering the emergence of *de novo* byproducts in the safety assessments for vaping liquids. Caution should be exercised not only with the large numbers of adolescents exposed to and addicted to vaping products but also adults who aim to benefit from a safer alternative to cigarettes. In the meantime, e-cigarette use, especially those containing flavorings, should be discouraged in the younger population and regulations revised while product safety is being thoroughly examined.

## Experimental Section

### Surfactant Models

The SLS mixture was designed and developed from lipidomic analysis and literature review,^29,47^ and the final lipids and lipid ratios used aim to mimic the saturated, unsaturated, charged lipid, and neutral lipid composition of lung surfactant: 68:20:10:2 weighted ratio of DPPC:POPC:POPG:cholesterol.^44,45,64^ All lipids were sourced from Avanti Polar Lipids (USA) and dissolved to 1 mg/mL in 2:1 chloroform–methanol (Sigma-Aldrich, Germany). The bovine-derived clinical surfactant Alveofact (45 mg/mL, Lyomark Pharma, Germany)^60^ was diluted in 0.9% saline (pH 5.8, Sigma-Aldrich) to 1 mg/mL. The chemical to lipid ratio in alveolar physiological conditions is not known. Ratios employed in this study emulate previous studies.^15,39^

### Compression–Expansion Models

#### Langmuir–Blodgett Trough

The LBT (NIMA Technology Ltd., England) was custom-designed with a continuously enclosed Teflon-vitrified coated ribbon replacing classical barriers and filled with 0.9% saline (pH 5.8) at 25 °C. Surface pressure was quantified *via* a Wilhelmy cellulose plate to produce surface pressure–area (Π–*A*) isotherms (Supporting Information Figure S6). The LBT was operated *via* NIMA software. To ensure LBT accuracy, DPPC Π–*A* isotherms were reproduced regularly as systematic controls according to the literature.^32^ As an additional control, before each test, a saline (Π–*A*) isotherm was produced to verify a constant surface pressure of 0 mN/m, *i.e.* the surface pressure of water. Thereafter, 20 μL of SLS was deposited at the air–liquid interface with a Hamilton gastight syringe (Hamilton Company, U.S.A), before a 5 min equilibration period to allow monolayer self-assembly. This was followed by ten compression–expansion cycles at 150 cm^2^/min, moving between 215 cm^2^ area and 56 cm^2^. Alternatively, after 5 min, BA (no. 418099, Sigma-Aldrich) or BPGA (no. W213000, Sigma-Aldrich), diluted to 1 mg/mL in 2:1 chloroform–methanol, was added at a chemical to lipid molar ratio of 1:10 prior to each experiment. Three independent replicates were collected for each condition. Data were extracted from NIMA software, which records the surface area (*A*, cm^2^) and surface pressure (Π, mN/m). Changes in area were recorded as relative changes from the total surface area of our trough (≈230 cm^2^) to enable field-wide isotherm comparisons from other troughs.

#### Constrained Sessile Drop Surfactometer

A custom-designed constrained sessile drop system was employed. This system uses elements of the CSD surfactometer from Krüss and its software (Drop Shape Analyzer). The Krüss Advance Software calculates surface tension *via* the contact angle between the sessile drop and pedestal (Supporting Information Figure S6B), allowing the production of γ–*A* iso-cycles. The custom-built pedestal made of stainless steel was adapted to fit the equipment (Krüss, Germany). A microsyringe (ILS, Germany) was connected to a stepper motor computer-controlled system to produce finely regulated physiological compression–expansion cycles. Drop formation, recording, and analysis were performed *via* recordings from an UI-3060CP Rev. Two camera (IDS, Germany). Drops consisted of 0.9% saline with a volume of 12–14 μL. Oscillations were designed to mimic physiological conditions, meaning up to 20% reduction of surface area and a rate of up to 20 cycles per minute.^50,65^ Each replicate was run for 120 s, with a recording rate of 5 frames per second. Cycles were completed at room temperature (25 °C). For each replicate, saline was run alone to ensure approximately 72 mN/m surface tension before adding 1 μL of SLS or 3 μL of Alveofact to the air–liquid interface with a Hamilton pipet. These quantities were determined by selecting the volume necessary to reach the γ_min_ possible for each model (approximately 3 and 7 mN/m, respectively). When required, 100 μg/mL BA or BPGA was added immediately after the surfactant at a chemical to lipid molar ratio of 1:10. Alveofact weighted average molecular weight was estimated based on the known major lipid components (Lyomark Pharma, Germany). Drops were left for 5 min prior to initiating compression–expansion cycles. Three independent replicates were gathered for each condition.

### Parameters Characterizing the Biophysical Function of the Surfactant

#### Minimum Surface Tension (γ_min_)

Surface pressure is a direct correspondent of surface tension (both have units of mN/m), *via* the equation Surface pressure (Π) = Surface tension of water (γ_0_) – Surface tension (γ). The surface tension of water at a 20 °C air–water interface is 72.8 mN/m,^66^ meaning that at the minimum surface tension (γ_min_) of 0 mN/m, surface pressure is at its maximum (Π_max_) of ≈72 mN/m. Changes in (γ_min_) and/or (Π_max_) inform of surfactant functionality and stability.^67^ (γ_min_) or (Π_max_) for each cycle was extracted from exported CSD or LBT data, respectively.

#### Compressibility Modulus (κ)

In the surfactant context, the compressibility modulus (κ, mN/m) is a measure of resistance to lateral compression of a lipid film. A higher compressibility modulus corresponds to a compact lateral self-assembly, thus low film elasticity.^68^ It is defined as
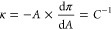
1with *C* being the compressibility sometimes reported in monolayer studies.

This theory was applied to the first LBT Π–*A* isotherms using Origin software (OriginLab, USA). Savitsky–Golay 9-point smoothing was then employed for improved visualization. For CSD data, the number of data points in each iso-cycle was insufficient for comprehensive compressibility moduli; rather, a global compressibility was estimated by calculating the slope between minimum to maximum iso-cycle points (Δγ/Δ*A*).^69^

#### Hysteresis

High hysteresis values describe a refinement or loss of material between compression and expansion, altering lateral lipid organization.^30^ In this study, hysteresis was represented by the difference in area (Δ*A*, cm^2^) between compression and expansion isotherms at half of the maximum surface pressure (LBT) or tension (CSD). For LBT data, hysteresis values from the first and last cycles were collected. Due to the large cycle numbers in CSD data, this data set increased to the mean of the first and last three cycles for more representative analysis.

## Atomistic Molecular Dynamics Simulations

We performed two sets of atomistic molecular dynamics simulations to characterize the interaction of the vaping chemicals on the pulmonary surfactant monolayers.

### Static Simulations of Protein-Free Monolayers

First, protein-free surfactant monolayers were simulated at 11 fixed areas per lipid, ranging from 50 to 100 Å^2^ with 5 Å^2^ intervals and thus covering the physiologically relevant compression states. The simulation contained two monolayers separated on one side by a slab of ≈27,040 water molecules (≈91 per lipid) with ≈150 mM NaCl and on the other side by a large slab of vacuum. The two monolayers each contained a total of 149 lipids with molar ratios of 68/20/10/2 of DPPC/POPC/POPG/cholesterol, *i.e.*, in accord with the experimental SLS mixture. The simulations were performed with either low or high concentrations of BA (32 or 320 molecules) or BPGA (20 or 200 molecules). A vaping-chemical-free system was simulated as a control. All simulations were 1 μs long.

The amount of L_c_-like packing was characterized by clustering the 10th carbon atoms along the acyl chains of phospholipids and the C14 atom of cholesterol in the plane using the DBSCAN algorithm with a cutoff of 0.71 nm and a requirement for 6 neighbors within this cutoff.^44^ Any acyl chain or cholesterol molecule that was found to be part of a tightly packed cluster was assigned to the L_c_ phase, and the total fraction of this phase was then averaged over time for each static simulation.

The contact preferences with lipids were calculated using the gmx mindist tool bundled with GROMACS. Only non-hydrogen atoms were included to speed up the analysis, and the cutoff was set to 0.6 nm. All contacts were normalized based on the number of possible interaction partners.

The density profiles were calculated using the gmx density bundled with GROMACS. The profiles were centered at the phosphate position, and all profiles were normalized to have a maximum value of 1.

The tilt of the acyl chains was averaged over all phospholipids, over their two chains, and over time. The tilt angle was defined as the angle between the vector connecting the first and 16th carbons of the acyl chains and the normal to the monolayer (*z* axis).

### Dynamic Simulations with Surfactant Proteins

Second, we performed dynamic simulations of the pulmonary surfactant monolayers in which the monolayer was compressed so that the APL decreased from 110 to 54.5 Å^2^ in the course of a 2 μs-long simulation. The monolayer composition was 60/20/10/10 of DPPC/POPC/POPG/cholesterol, following our earlier work^44,45^ and hence slightly different from the SLS mixture used in experiments and in the static simulations. Two monolayers present in the simulation system were again separated by a slab of water (38,400 molecules, ≈75 per lipid with ≈150 mM NaCl) on one side and vacuum on the other side (across the periodic boundary conditions). The monolayers contained either only lipids or a single copy of SP-B or SP-C each.^45^ Each system was simulated in the absence of vaping chemicals as well as in the presence of 320 molecules of BA or 200 molecules of BPGA. The simulations were performed in duplicate.

From the dynamic simulations, we analyzed the numbers of contacts between the vaping chemical and surfactant proteins, as well as the lipids. This analysis was performed over the dynamic trajectory. The two replica simulations were analyzed, and their mean values and differences were used as the reported result and its error estimate, respectively. Hydrogens were omitted from the analysis, and the numbers were normalized based on possible interaction partners present in the simulation. A cutoff of 0.6 nm was used to define a contact.

### Force Fields and Simulation Parameters

We used the CHARMM36 model to describe the lipids^70,71^ and the 4-point OPC water to model water.^72^ This force field combination has successfully captured the interfacial physics of the water–air interface and monolayers placed thereon.^44,55,56^ The vaping chemicals were described with the Merck molecular force field^73^ obtained from SwissParam.^74^ For proteins, the CHARMM36m force field^75^ was used with the protein models adapted from our previous work.^45^

For the static simulations with a fixed monolayer area, the simulation protocol followed our earlier work^44^ except that the vaping chemicals were originally placed in the aqueous phase. For the dynamic simulations with a slowly changing monolayer area, we also followed our earlier work^45^ apart from the presence of the vaping chemicals.

Simulation inputs and outputs are openly available in the Zenodo repository at DOIs: 10.5281/zenodo.10451123 and 10.5281/zenodo.10451559.

### Statistical Analysis

Where appropriate, two-way analysis of variance (ANOVA) tests were performed, followed by Dunnett’s multiple comparisons tests to compare every group mean to the control. Significance was determined with the alpha set to 0.05. The standard error measurement was calculated and presented as error bars where suitable. All graphs and statistical analyses for *in vitro* surfactometer work were produced utilizing GraphPad Prism 9.1.0 Software (GraphPad, USA).

## Supporting Information Available

Representative Π–*A* iso-cycles for SLS with BA or BPGA; SLS LBT temperature experiments; vaping chemical surface activity; clinical surfactant Alveofact Π–*A* isotherms; CSD surface tension–time cycles; overview of LBT and CSD techniques; snapshots of simulations with protein-free monolayers and the lower concentration of the vaping chemicals; and snapshots of SP-B interacting with BPGA (PDF)
