# Supplementary material for: Exposure to Aldehyde Cherry e-Liquid Flavoring and Its Vaping Byproduct Disrupt Pulmonary Surfactant Biophysical Function
Source: Environ Sci Technol. 2024 Jan 8;58(3):1495–508. doi: 10.1021/acs.est.3c07874 (PMC10809783; doi:10.1021/acs.est.3c07874)
Supplement: Supplementary file 1 — es3c07874_si_001.pdf [file es3c07874_si_001.pdf]

Supporting Information: Exposure to Aldehyde Cherry e-Liquid Flavoring and Its Vaping Byproduct Disrupt the Pulmonary Surfactant Biophysical Function

Alexia Martin,[†] Carmelo Tempra,[‡] Yuefan Yu,[†] Juho Liekkinen,[¶] Roma Thakker,[†] Hayoung Lee,[†] Berta de Santos Moreno,[†] Ilpo Vattulainen,[¶] Christos Rossios,[†] Matti Javanainen,[§,‡] and Jorge Bernardino de la Serna[*,†]

†*National Heart and Lung Institute, Imperial College London, Sir Alexander Fleming Building, London, SW7 2AZ, U.K.*

‡*Institute of Organic Chemistry and Biochemistry, Czech Academy of Sciences, Prague 6, 160 00, Czech Republic*

¶*Department of Physics, University of Helsinki, Helsinki, 00560, Finland*

§*Institute of Biotechnology, University of Helsinki, Helsinki, 00790, Finland*

E-mail: j.bernardino-de-la-serna@imperial.ac.uk

Phone: +44 (0)20 75943277

This supporting information document consists of 8 pages and contains 8 figures (S1–S8).

Supplementary Methods

SLS LBT Temperature Experiments

SLS (outlined in main methods) was trialed on the LBT at 25°C as well as 37°C, maintained with a TC120 water bath (Grant Instruments, UK), where both 20 µL and 35 µL volumes of 1 mg/ml SLS were used in attempts to reach Π_{max} of 72 mN/m. SLS was deposited onto the air–liquid interface with a Hamilton gas tight syringe (Hamilton Company, U.S.A), before being left to adsorb to the interface for five minutes prior to proceeding with compression at a rate of 150 cm^2/min, moving from maximum area 215 cm^2 to minimum area 56 cm^2.

Alveofact LBT Trials

When using Alveofact (Lyomark Pharma, Germany) on the LBT, 20 µL 1 mg/ml was initially run on the LBT with similar procedure to SLS. In attempts to reach higher surface pressures, Alveofact concentration was increased to 10 mg/ml (diluted in 0.9% saline, Sigma-Aldrich) and added in volumes of 4 µL and 10 µL (2× and 5× molar quantities, respectively).

Vaping Chemical Surface-activity

BA and BPGA were tested alone on the CSD model. BA (no.418099, Sigma-Aldrich, Germany) and BPGA (no.W213000, Sigma-Aldrich) were serially diluted to 1 mg/ml from 1 g/ml in factors of 10 in 2:1 chloroform-methanol (Sigma-Aldrich). After first running saline alone, 1 µL of each concentration was added to the drop and run for 60 s at 20 cycles per minute consecutively in ascending order without prior surfactant addition.

Supplementary Results

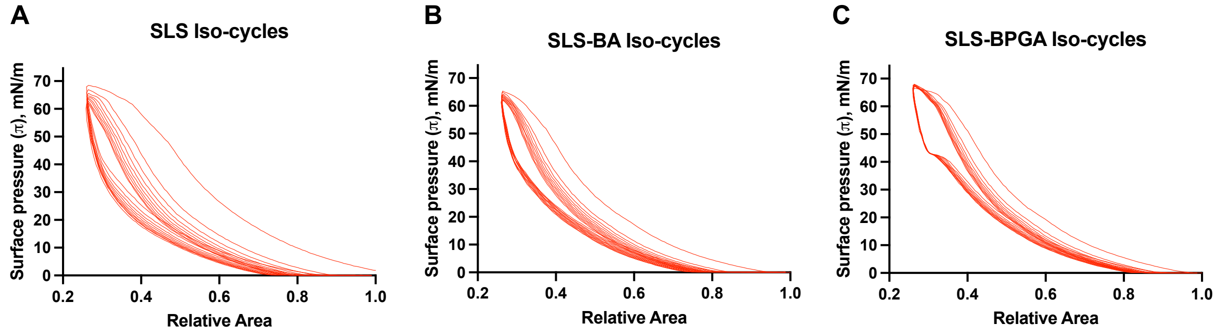

Figure S1: **Representative $\Pi - A$ Iso-Cycles for SLS with BA or BPGA**. A) SLS alone. B) SLS with BA at a 1:10 molar ratio to surfactant lipids. C) SLS with BPGA at a 1:10 molar ratio to surfactant lipids. Cycles were completed on a saline subphase at 25°C with a compression rate of 150 cm^2/min over ten cycles. Three independent replicates were collected, a representative replicate from each condition is presented for clarity.

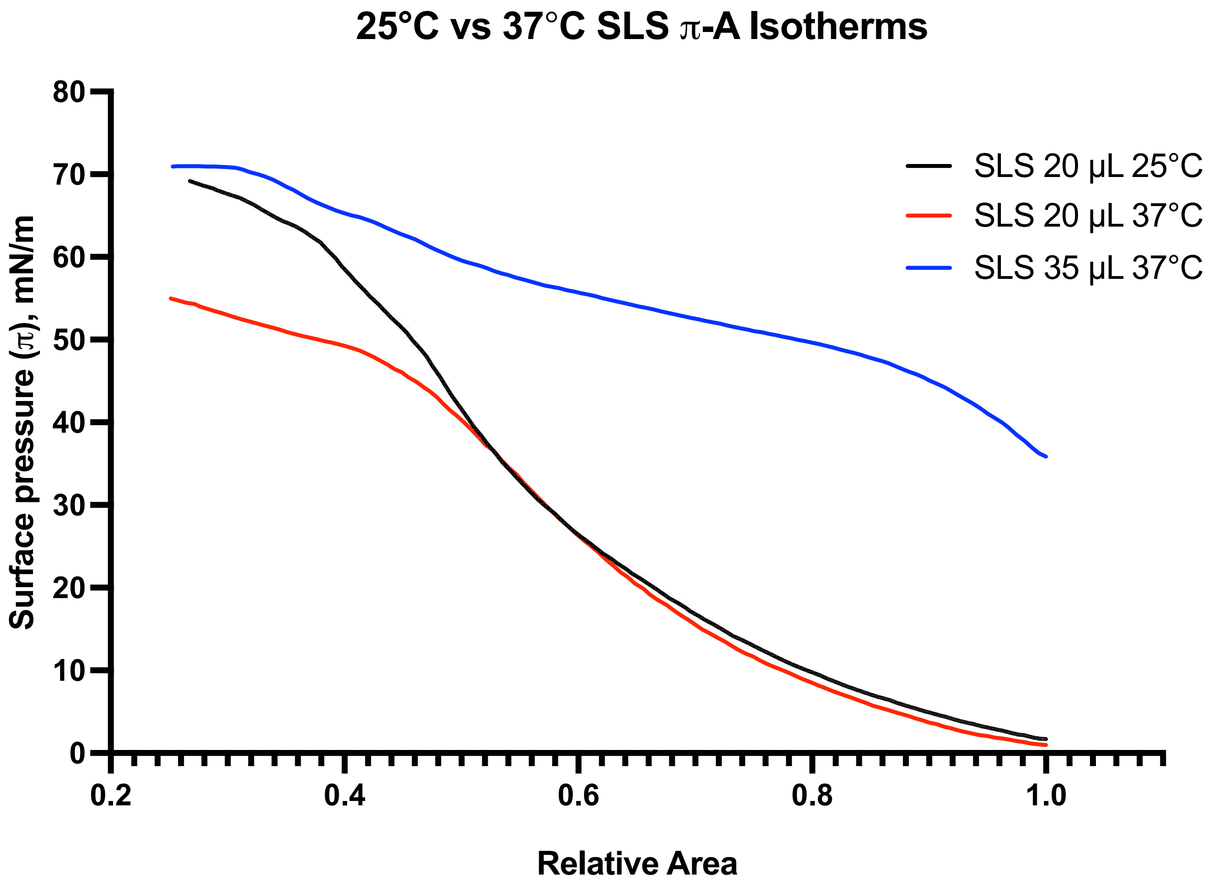

Figure S2: **SLS LBT temperature experiments.** A 25°C $\Pi - A$ isotherm is compared to an isotherm at 37° also produced with 20 µL 1 mg/ml SLS, and an isotherm produced with 35 µL volume. Isotherms were completed on a saline subphase at 25° with a compression rate of $\Pi - A$. Three independent replicates for each condition.

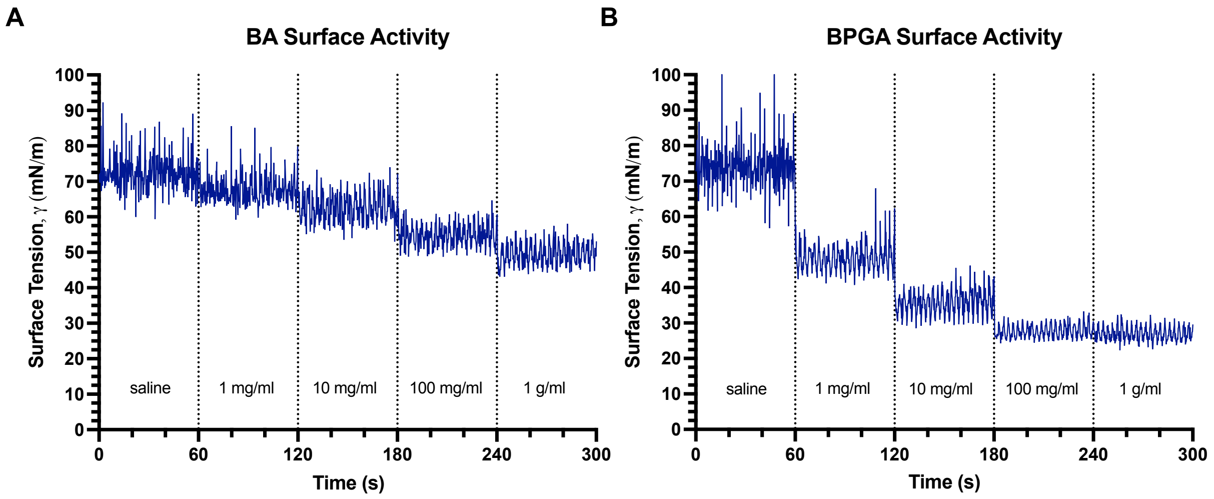

Figure S3: **Vaping chemical surface activity.** A) BA surface activity. B) BPGA surface activity. For both chemicals, concentrations were added to the CSD in ascending order, 1 µL at a time, after each undergoing compression–expansion cycles for 60 seconds. Three independent replicates, for clarity a representative replicate is shown in the figure.

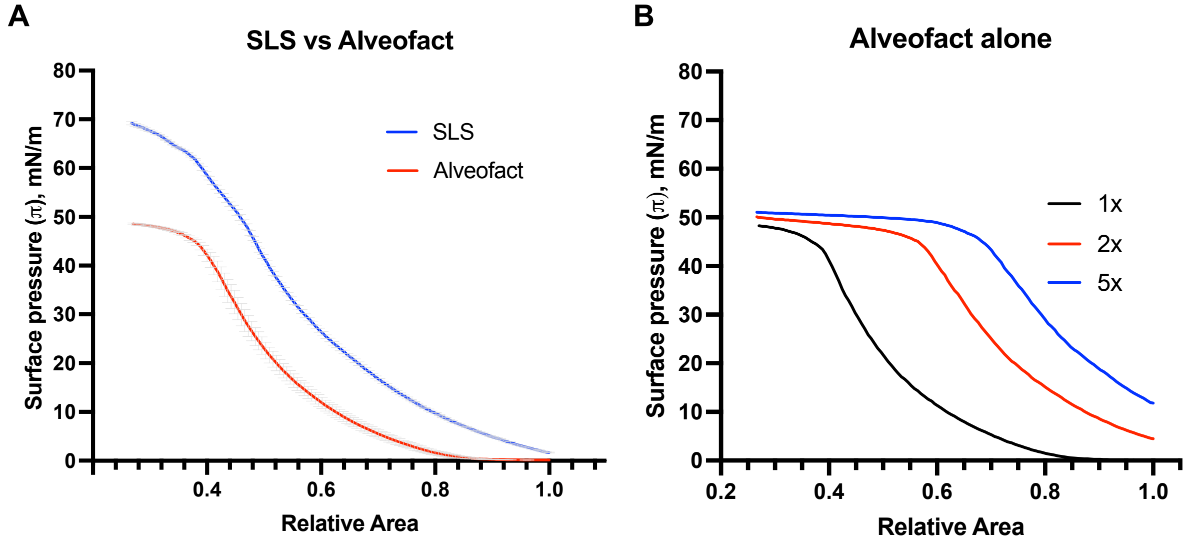

Figure S4: **Clinical surfactant Alveofact $\Pi - A$ isotherms.** A) SLS vs Alveofact on the LBT. $\Pi - A$ isotherms run with 20 µg in chloroform-methanol of each surfactant at 150 cm^2/min on a 25°C saline subphase. Error bars represent standard error. Three independent replicates were produced for each condition. B) $\Pi - A$ isotherms for a range of Alveofact quantities. "1x" refers to 20 µL 1 mg/mL Alveofact. "2x" and "5x" refer to two times and five times molar quantity, respectively. For clarity, representative isotherms are shown for each condition.

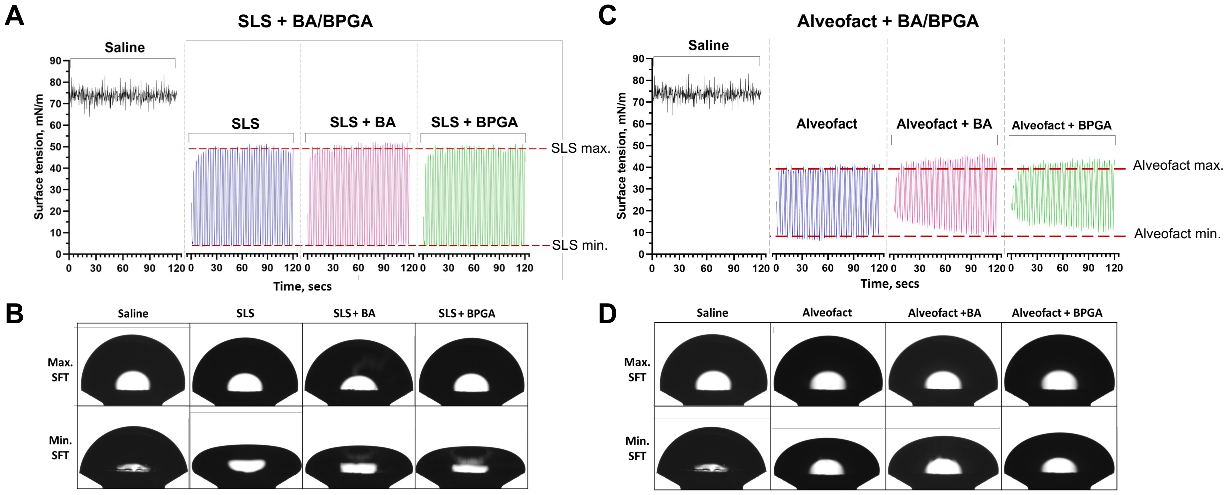

Figure S5: **CSD surface tension–time cycles.** A) SLS CSD compression-expansion cycles with BA and BPGA. Red lines outline the SLS alone maximums and minimums. B) Photographic representation of the Sessile Drop at maximum and minimum surface tension of the last cycle with conditions in A). C) Alveofact CSD compression-expansion cycles BA and BPGA. Red lines outline the Alveofact alone maximums and minimums. D) Photographic representation of the Sessile Drop at maximum and minimum surface tensions of the last cycle with conditions in C. The saline drop underwent cycles alone prior to the addition of any surfactant or vaping chemical. Cycles were completed at a rate of 20 cycles per minute over two minutes. Three independent replicates were collected for each condition.

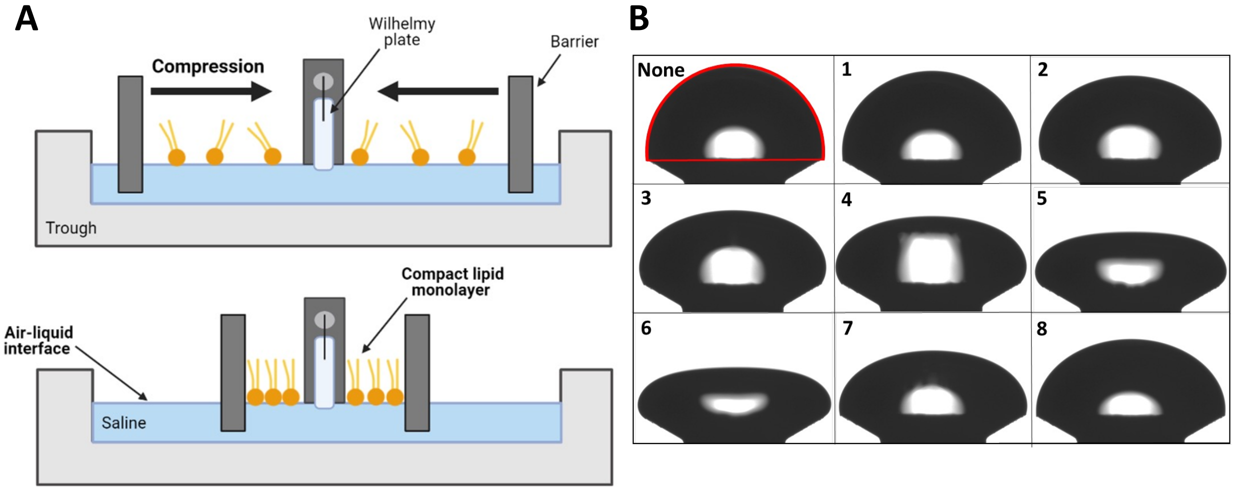

Figure S6: **Overview of LBT and CSD techniques.** A) A diagrammatic representation of the LBT set-up and $\Pi - A$ isotherm production. A surfactant film sitting at the air-liquid interface is compressed by reducing surface area via movement of motorised ribbon barriers to form a compact lipid monolayer. Surface pressure is recorded via the Wilhelmy plate surface pressure sensor and NIMA Software. B) Photographic representation of a CSD compression-expansion cycle. The drop, outlined in red, is sat on a stainless-steel pedestal (None). Surfactant can then be added and made to undergo compression-expansion cycles at physiological rates (1-8). The drop is compressed and expanded via the automated removal and replacement of subphase from beneath the pedestal. Surface tension is calculated from the contact angle between the pedestal and the drop surface in each image.

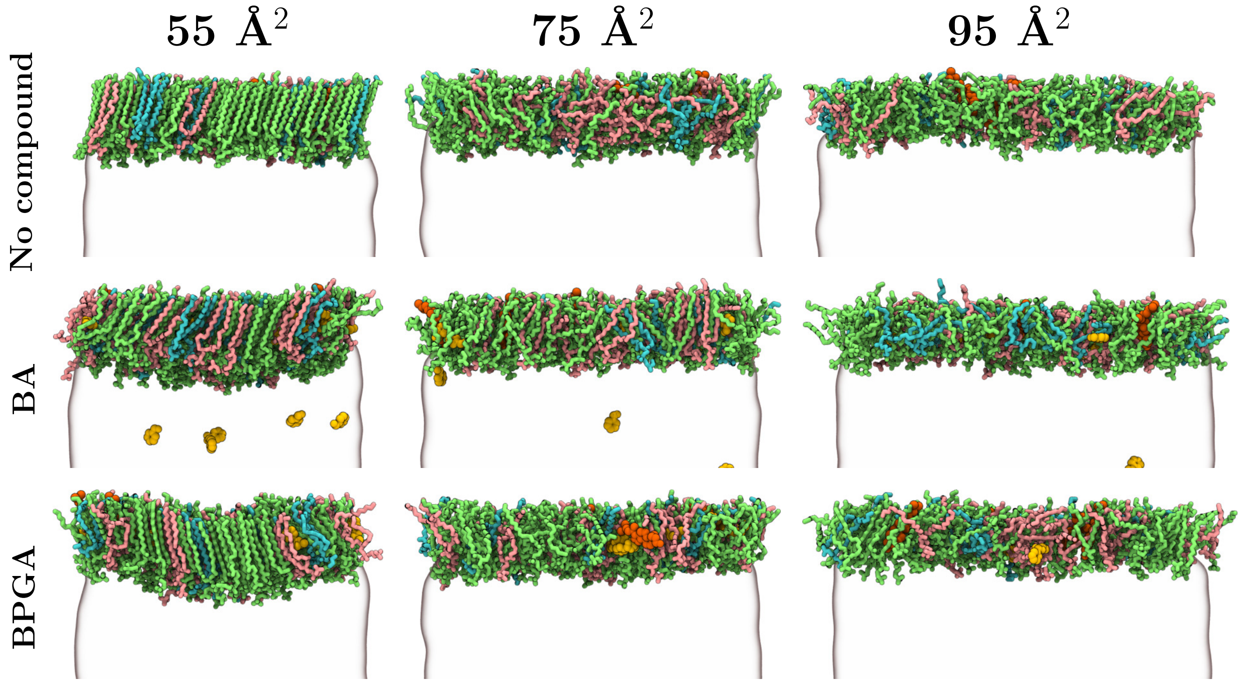

Figure S7: **Snapshots of the simulations with protein-free monolayers and the lower concentration of the vaping chemicals.** Coloring as in Fig. 2.

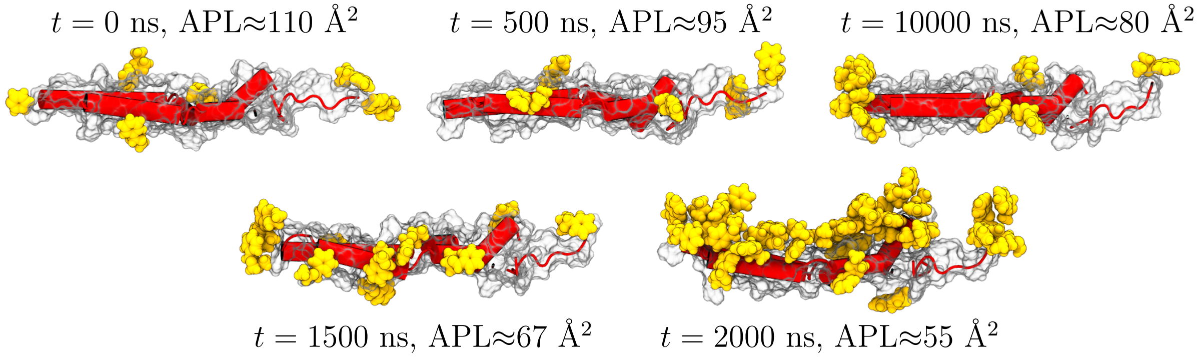

Figure S8: **Snapshots of the SP-B interacting with BPGA.** Coloring as in Fig. 5. The gaseous (aqueous) phase is towards the top (bottom) of the page.
